# The Objective Dementia Severity Scale Based on MRI with Contrastive Learning: A Whole Brain Neuroimaging Perspective

**DOI:** 10.3390/s23156871

**Published:** 2023-08-02

**Authors:** Yike Zhang, Wenliang Fan, Xi Chen, Wei Li, on behalf of the for Alzheimer’s Disease Neuroimaging Initiative

**Affiliations:** 1 School of Artificial Intelligence and Automation, Huazhong University of Science and Technology, Wuhan 430074, China; 2 Image Processing and Intelligent Control Key Laboratory of the Education Ministry of China, Wuhan 430074, China; 3 Department of Radiology, Union Hospital, Tongji Medical College, Huazhong University of Science and Technology, Wuhan 430074, China

**Keywords:** medical robots, disease evaluation, Alzheimer’s disease, contrastive learning

## Abstract

In the clinical treatment of Alzheimer’s disease, one of the most important tasks is evaluating its severity for diagnosis and therapy. However, traditional testing methods are deficient, such as their susceptibility to subjective factors, incomplete evaluation, low accuracy, or insufficient granularity, resulting in unreliable evaluation scores. To address these issues, we propose an objective dementia severity scale based on MRI (ODSS-MRI) using contrastive learning to automatically evaluate the neurological function of patients. The approach utilizes a deep learning framework and a contrastive learning strategy to mine relevant information from structural magnetic resonance images to obtain the patient’s neurological function level score. Given that the model is driven by the patient’s whole brain imaging data, but without any possible biased manual intervention or instruction from the physician or patient, it provides a comprehensive and objective evaluation of the patient’s neurological function. We conducted experiments on the Alzheimer’s disease Neuroimaging Initiative (ADNI) dataset, and the results showed that the proposed ODSS-MRI was correlated with the stages of AD 88.55% better than all existing methods. This demonstrates its efficacy to describe the neurological function changes of patients during AD progression. It also outperformed traditional psychiatric rating scales in discriminating different stages of AD, which is indicative of its superiority for neurological function evaluation.

## 1. Introduction

Alzheimer’s disease (AD) is a progressive neurodegenerative disease with an insidious onset. It is the most common cause of dementia and one of the most expensive and deadliest diseases of the century [1]. Clinical manifestations include memory impairment, aphasia, apraxia, agnosia, impairment of visuospatial skills, executive dysfunction, personality changes, and behavioral changes. The cause of AD is still unclear; however, its progression is usually accompanied by a decline in neurological function. Therefore, in clinical diagnosis, physicians attempt to evaluate subtle changes in the progression of the disease stage by evaluating the level of neurological function and recommending an appropriate treatment plan. The Psychiatric Rating Scale is one of the auxiliary evaluation methods commonly used by physicians. There are many kinds of psychiatric rating scales for Alzheimer’s disease; however, these scales are not sufficiently accurate and comprehensive, which leads to the low credibility of the traditional scales. Moreover, they lack objectivity and are not a reliable basis for clinical diagnosis and treatment. Structural magnetic resonance imaging (sMRI) is another auxiliary evaluation method that allows physicians to diagnose based on the degree of brain atrophy in MRI images. However, this approach is highly dependent on the physician’s subjective judgment and does not provide a detailed quantitative evaluation.

Deep learning is a powerful method that has been applied to medical images and automated accomplishing targeted tasks in an end-to-end way. There have been many published studies in recent years that have successfully applied this approach to medical image analysis tasks and generally achieved significant improvements [2,3,4]. Most previous studies focused on the prediction of disease categories, classifying patients’ images into discrete categories. However, for many types of diseases, the stages may exhibit a continuous variation in severity that can change over time [5,6,7]. These different stages of a disease are often classified into ordinal levels (e.g., normal, mild, moderate, and severe), but variations within these ordinal levels are usually underappreciated, and deep learning methods have been applied to evaluate disease stages in limited cases. This may be owing to the absence of the gold standard and representative samples for determining the AD stage.

In the field of deep learning, evaluating the differences between images can be expressed as a metric learning problem. The contrastive learning strategy is commonly employed for metric learning, and it is often used to evaluate the similarity between images. This strategy trains a network using contrastive loss functions and considers the metric between the network output vectors as the evaluation criterion for the image similarity metric. Therefore, we utilized a contrastive learning strategy to evaluate the sMRI images of patients with AD to complete the disease stage discrimination.

In this paper, an evaluation model is proposed based on contrastive learning to evaluate the neurological function of patients with AD. The evaluation model takes the sMRI images of the patient’s brain as the network input, uses a twin residual neural network as the network backbone, and trains the network with a supervised contrastive loss function. The supervised contrastive learning loss function effectively utilized the medical image information of patients with AD, as well as their label information. In the evaluation phase, we abstract the neurological level difference into the vector distance calculated from the twin neural network to obtain the neurological function score of patients with AD.

The main contributions of this paper are as follows:

(1) We propose an objective dementia severity scale based on MRI (ODSS-MRI) using a contrastive learning framework to evaluate neurological function during AD progression, which is driven by the whole brain image of the patient. This effectively ensures that the evaluation score will not be affected by any possible biased subjective factors during the testing of the physician or patient.
(2) The score obtained from the proposed ODSS-MRI is highly correlated with the stages of AD, indicating that ODSS-MRI can accurately describe the neurological function changes during AD progression.
(3) The ODSS-MRI scores perform much better on discriminating different stages of AD progression compared to traditional psychiatric rating scales, indicating its advantage in the neurological function evaluation of AD.

## 2. Related Work

In this section, we briefly introduce the current methods for the evaluation of the neurological function in AD and contrastive learning methods related to the evaluation proposed in this paper.

### 2.1. Evaluation of Neurological Function in Alzheimer’s Disease

Due to the continuous growth of the aging population in China, the number of patients with AD is increasing, and an accurate and refined disease stage evaluation of AD has become a critical problem in clinical treatment. Clinically, AD begins with lesions on the brain. However, patients often do not notice any associated changes initially, and only after years of accumulation do patients exhibit obvious symptoms. Moreover, doctors cannot simply use medical imaging and clinical diagnostic data to diagnose the patient’s real situation. Usually, physicians evaluate the neurological function of the patients to diagnose the stage of AD. Currently, one of the main methods for this purpose is neuropsychological testing, which allows physicians to measure the neurological function of patients by observing their performance in tasks involving multiple brain functions, such as movement, language, attention, memory, and thinking, and quantitatively evaluating their neurological function to obtain a score within a certain range [8,9,10,11,12,13]. The common traditional neuropsychological tests include the Mini-Mental State Examination (MMSE) [8], Clinical Dementia Rating (CDR) [9], Functional Activities Questionnaire (FAQ) [10], Alzheimer’s Disease Assessment Scale-Cognitive (ADAS-COG) [11], and Rey Auditory Verbal Learning Test (RAVLT) [12]. The MMSE primarily screens for cognitive impairment by quizzing both orientation and memory, whereas the CDR screens for mild cognitive impairment and classifies clinical dementia by quantifying life skills. Although neuropsychological tests can visually reflect different aspects of patients’ brain function, each scale suffers from the problem of incomplete evaluation of neurological function due to different emphases on neurological function evaluation, which in turn affects the diagnosis of the severity of the disease. At the same time, the traditional psychiatric rating scales utilize a question-and-answer format with cooperation between physicians and patients, which may lead to a lack of patient cooperation during the evaluation stage, and the score is also affected by the biased subjectivity of the physician.

With the rapid development of medical imaging technology in recent years, neuroimaging has gradually become one of the main methods to evaluate the neurological function of patients, with structural MRI being the most frequently used [14,15]. Structural MRI is a mature medical imaging technique, which has become another important method for physicians to evaluate the AD stage of patients because of its safety, non-invasiveness, reproducibility, and sensitivity to morphological changes in the brain. During AD, the patient’s brain atrophy gradually increases [16]. Based on this characteristic, physicians usually assess the patient’s condition based on the degree of brain atrophy and the volume of gray and white matter in the sMRI images; however, due to the individual differences of patients, it is difficult for physicians to accurately diagnose the disease progression of patients.

### 2.2. Alzheimer’s Diagnosis Based on Deep Learning

With the great popularity of deep learning, many researchers have tried to apply this approach to study the pathogenesis of AD, diagnose the stages of AD, and so on. For example, Suk et al. [17] combined MRI, PET, MMSE, and other data to train a classifier and reported promising experimental results in their paper; Ortiz et al. [18] achieved more than 90% accuracy on AD and Normal Control (NC) classification problems by training multiple classifiers followed by voting; Lian et al. [5] proposed a hierarchical fully convolution network to automatically identify local regions with the discriminatory ability in whole-brain sMRI, and on this basis, multi-scale feature representation was integrated to construct a hierarchical classification model for AD diagnosis.

So far, many studies have achieved good results on the classification problem of AD, but it is not enough to just solve the classification problem. The discrete label obtained by prediction lacks information on disease-stage changes. Few studies have applied deep learning methods to the problem of neurological function evaluation in AD; however, some studies use contrastive learning strategies to evaluate the severity of other diseases. For example, Li et al. [19] applied the contrastive learning strategy to continuous disease severity evaluation and disease change detection in retinopathy and osteoarthritis, and the output of the trained twin neural network achieved a correlation of more than 0.85 with the expert sort order on the 100-image test dataset ranked by experts; Li et al. [20] applied the contrastive learning strategy to the detection of psoriasis severity and located skin lesions in the image. The severity scores obtained by the trained network framework achieved better results than the severity index method commonly used in psoriasis. Contrastive learning has the potential to solve the problem of neurological function evaluation in AD. In general, deep learning has achieved better results than traditional methods in diagnosing the stage of AD; however, no deep learning method is available for the problem of neurological function evaluation in AD.

### 2.3. Contrastive Learning

Contrastive learning is a type of metric learning, which focuses on learning the common features between similar instances and distinguishing the differences between dissimilar instances [21,22,23,24,25,26,27]. Compared to traditional deep learning, contrastive learning does not need to focus on the details of instances but only needs to learn to distinguish data at the abstract semantic level of the feature space. Therefore, its optimization is simple, and the model can be more robust. The goal of contrastive learning is to train an encoder that can encode similar data of the same class while making the encoding results of different classes of data as different as possible. This approach can be divided into self-supervised contrastive learning and supervised contrastive learning depending on whether artificial annotations are used in the training stage.

Self-supervised contrastive learning is often used for network pre-training by constructing agent tasks to enable models to learn robust features of large-scale data without using human annotations [28,29,30,31]. Self-supervised contrastive learning constructs positive and negative sample pairs by data transformation so that the model learns to aggregate similar samples and mutually reject dissimilar samples in the feature space, thus aggregating similar samples together in the feature space. In recent years, many self-supervised contrastive learning algorithms have been proposed [21,22,23,24], and the models pre-trained using contrastive learning methods perform well on many downstream tasks, such as image classification, image segmentation, and face recognition.

Another approach in the contrastive learning domain is supervised contrastive learning. It was proposed in recent years by Khosla P et al. [25]. Supervised contrastive learning added artificial annotation information to the loss function based on self-supervised contrastive learning. Generally, in self-supervised contrast learning, an anchor sample has only one positive sample and all the other samples are regarded as negative samples. In the case of supervised contrastive learning, each anchor sample can have multiple positive and negative samples using labels. This incorporates the intra-class relationship of the data into the loss function, and in this way, the encoder can consider the intra-class similarity between the data during the training stage, rather than compressing similar data into one point of the feature space as in the self-supervised contrastive learning.

## 3. Materials and Methods

### 3.1. Materials

#### 3.1.1. Participants

The sMRI data used in this experiment were obtained from the ADNI program (adni.loni.usc.edu, accessed on 24 June 2022), which was established in 2003 and led by Principal Investigator Michael W. Weiner, MD. ADNI is a longitudinal multicenter study aimed at developing clinical, imaging, genetic, and biochemical biomarkers for the early detection and follow-up of AD. This program provides researchers with a variety of neuroimaging data, such as magnetic resonance imaging, functional magnetic resonance imaging, magnetic resonance diffusion tensor imagery, and positron emission scanning images, which is currently the most widely used public database of AD. We obtained 2997 sMRI images from 633 subjects in the ADNI dataset, including 149 AD, 263 mild cognitive impairment (MCI), and 221 NC subjects. Table 1 shows the demographic information of the subjects included in our dataset, including the category, number of subjects, sex (male/female), age (average), and the number of sMRI images.

#### 3.1.2. Data Pre-Processing

Data pre-processing was performed to ensure that the model was capable of effectively learning pattern information in the dataset. In the data-preprocessing stage, we obtained the image from the ADNI website after gradient nonlinear correction, non-uniform correction, and histogram sharpening. To quickly process a large amount of sMRI data, we used the Matlab-based CAT12 toolbox for batch processing of the data. First, we performed an anterior commissure-posterior commissure (AC-PC) correction of the image data and obtained standard brain atlas images using the AC-PC line as the baseline. Subsequently, we de-skulled and removed invalid areas of the images, preserving only the brain locations, after which we aligned the images with the Montreal Neurological Institute (MNI) standard template. Finally, we modulated the images to ensure spatial consistency and preserve variability.

After the pre-processing stage, we can obtain whole brain images with a size of 113 × 137 × 113. Subsequently, we sliced the 3D image. Considering that the middle part of the image contains richer brain information, we sliced the middle layer (69/137) of the 3D whole brain image along the coronal plane. Finally, we obtained the 2D slice dataset for this study and divided the dataset into the training dataset (80%) and the test dataset (20%) based on the subjects and added the image from the first patient participation test to the test dataset as historical data to the training dataset.

### 3.2. Methods

**Figure 1:**
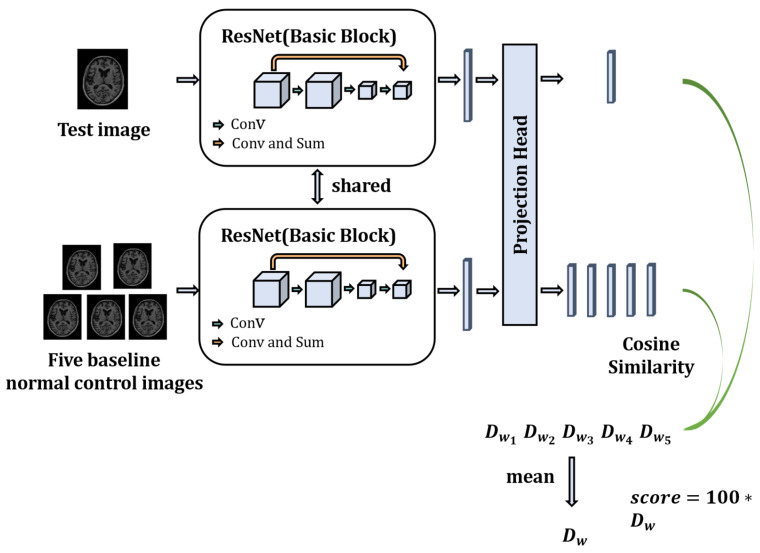
Schematic of the AD neurological function evaluation model based on contrastive learning. (∗ means multiplication).

In this section, we propose an objective dementia severity scale based on MRI using a contrastive learning strategy for AD neurological evaluation problems. The proposed evaluation model is driven by MRI and uses a contrastive learning framework to evaluate the neurological function of patients with AD. The model comprehensively considers the neurological functional information of the patient’s whole brain and does not require any additional knowledge of neurology and pathology, effectively ensuring that the obtained functional scores are not influenced by biased subjective factors of the physician and the patient. The model structure is shown in Figure 1. The model utilizes a two-channel structure, in which the test sample is to be evaluated. The MRI images of the healthy person were randomly selected as the baseline sample. They are utilized as input of two channels, and then the samples are entered into a Residual Networks (ResNet) model with shared weights to obtain the high-dimensional feature vectors of the sample to be evaluated and the baseline healthy person sample. We then map the high-dimensional feature vectors into a low-dimensional feature space using a Projection Head. Finally, the cosine similarity is used to calculate the similarity between the low-dimensional feature vectors of the sample to be evaluated and the baseline healthy subjects. The similarity is expanded by a hundredfold to obtain the final neurological function evaluation score.

1. Main structure of the model

**Figure 2:**
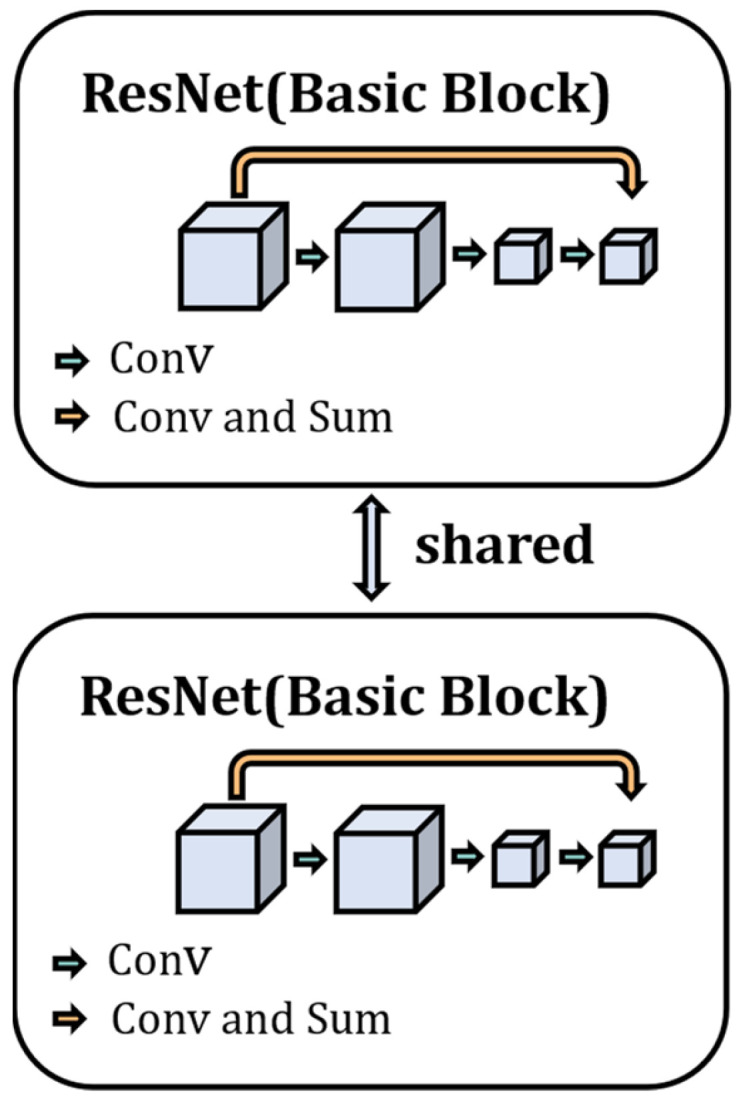
Schematic of the residual neural network with weight sharing.

The two-channel structure of the ODSS-MRI model is a weight-sharing residual neural network, as shown in Figure 2. First, the network simultaneously mines potentially valid pattern information from the data in both input channels and performs weight sharing during optimization to ensure that the samples in the two channels are mapped into the same feature space. Second, the model adapts the residual neural network as the feature encoder, which utilizes a skip connection, as shown in Figure 2, so that gradient diffusion does not occur when the depth of the network increases. At the same time, the network parameters are easier to optimize, and the gradient information propagates more easily during the back-propagation stage.

2. Model Training

**Figure 3:**
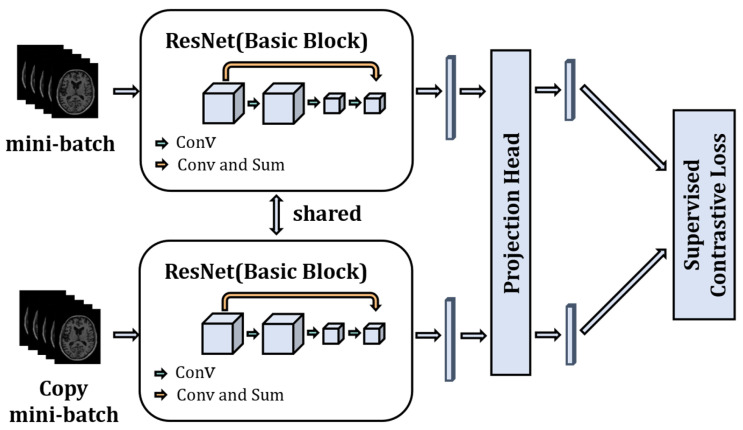
Schematic of the model training stage.

The model is trained using the supervised contrastive strategy. As shown in Figure 3, there are two channels, and each batch of images is copied once and fed into the two channels. Then the ResNet model is used as a feature encoder to extract features from the input images. ResNet uses the network structure of skip connection to significantly increase the depth of the network, which can extract semantically informative features from images. The fully connected layer is removed from ResNet and only the 1024-dimensional feature vector output obtained from the average pooling layer is used. A Projection Head is added after the encoder, which maps the 1024-dimensional feature vector space to a 128-dimensional feature vector space, and then we normalize the 128-dimensional feature vectors to keep them on a unit hypersphere. The essence of the projection head is a multi-layer perceptron consisting of linear fully connected layers and the ReLU function to add nonlinearity, and the number of fully connected layers of the projection head is set to one in the model. The study [24] (SimCLR) has shown that the addition of a learnable nonlinear transformation between the feature representation of the image and the contrastive loss can significantly improve the quality of the learned representation feature vector of the images. Finally, the supervised contrastive loss is used to train the model to fully utilize the image and label information of the data.

3. Supervised contrastive learning loss function

In the contrastive learning domain, the purpose of the loss function is to allow the model learning to cluster similar samples in the feature space and mutually exclude dissimilar samples so that similar samples are aggregated together in the feature space. Most contrastive loss functions do not use the label information, but supervised contrastive learning adds label information to the loss function based on contrastive learning, as shown in Equations (1) and (2), where *N* represents the amount of image data in a mini-batch, *y* represents the label of the image, *z* represents the feature vector, and *τ* represents the temperature parameter.

(1) $$L_{SupCon}=\sum_{i=1}^{2N}L_{supcon}^{i}$$

(2) $$L_{supcon}^{i}=-\frac{1}{2N_{y_{i}}-1}\overset{2N}{\underset{j=1}{\sum }}{Ι}_{i\neq j}*{Ι}_{y_{i}\neq y_{j}}*log\frac{\exp\left(z_{i}*\frac{z_{j}}{\tau }\right)}{\sum_{k=1}^{2N}{Ι}_{i\neq k}*\exp\left(z_{i}*\frac{z_{k}}{\tau }\right)}\;$$

The supervised contrastive loss function is a generalization of the contrastive loss function. Supervised contrastive loss treats data with the same label as positive pairs, and those with different labels as negative samples. This expands the number of positive pairs while maintaining a sufficient number of negative pairs. The loss function calculates the similarity for all positive pairs and then performs a weighted average, which contrasts with a large number of negative pairs. In addition, by increasing the number of positive samples, the network can better characterize intra-class similarities.

4. Neurological function scoring

After training the model, a distance metric function needs to be chosen to calculate the distance between the two output vectors, in order to transform the output feature vectors into neurological function scores. There are many distance metric functions, such as Euclidean distance and Mahalanobis Distance. However, the more similar the images in this framework, the higher the similarity of the two vectors corresponding to the output, and the smaller the distance between the images. Therefore, this framework utilizes the cosine distance to measure the distance between the two vectors. The neurological function score is calculated using Equation (3):

(3) $$score=100*\frac{z_{i}*z_{j}}{\max\left(\left|z_{i}\right|,\left|z_{j}\right|\right)}\;$$

**Figure 4:**
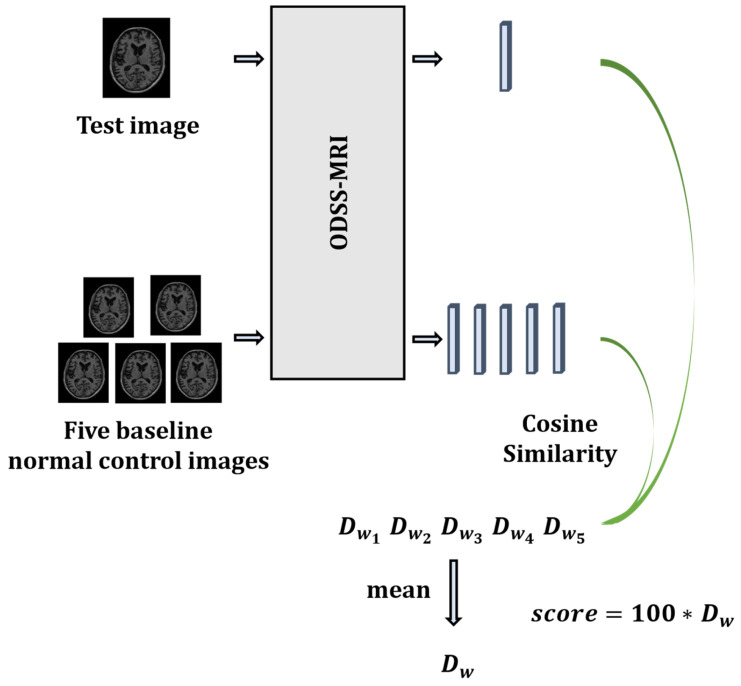
Neurological function scoring. (∗ means multiplication).

Figure 4 shows the specific process of neurological function scoring. We randomly select five baseline healthy individuals from the training dataset as the baseline for comparison with the test dataset. This approach assumes that healthy individuals exhibit a relatively consistent level of neurological function. However, given that subtle differences may exist between healthy individuals’ nervous systems, they are eliminated by averaging the five scores obtained.

## 4. Results

This section compares the ODSS-MRI with the traditional Psychiatric Rating Scales and analyzes the results.

### 4.1. Correlation Analysis

In the first part of this experiment, we compared the correlation between ODSS-MRI, traditional psychiatric rating scales, and the APOE allele gene with disease stage, and the results are shown in Table 2. The results showed that the scores obtained using the proposed ODSS-MRI have a Pearson correlation of 88.55% and a Spearman correlation of 87.12% with the stage of AD, which were 17.74% and 1.35% higher than CDR with the best performance in the traditional mental rating scale, respectively. This indicated that the ODSS-MRI can better describe the neurological function changes during AD progression compared to traditional psychiatric rating scales. The correlation between the NPI-Q, the GDS scale, and the APOE allele with the disease stage was low, ranging from 20% to 40%, which is far lower than the ODSS-MRI, and cannot be used as a reliable basis for identifying the disease stage of the subject.

**Figure 5:**
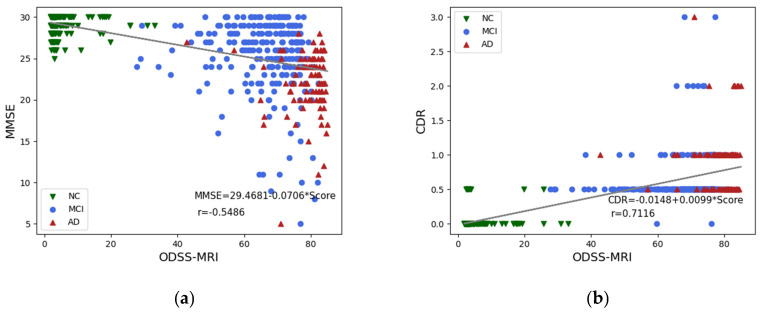
ODSS-MRI score and the MMSE (**a**) and CDR (**b**) scale score scatter plot. (∗ means multiplication).

The second part of the experiment examined the correlation between ODSS-MRI and the MMSE/CDR, as shown in Figure 5. The abscissa of the two scatterplots is the ODSS-MRI and the ordinate is the MMSE and CDR scale scores, respectively. On this basis, we used the ODSS-MRI to fit the MMSE and CDR scale scores. The experimental results showed that the ODSS-MRI model score was negatively correlated and positively correlated with the MMSE and CDR scores, respectively, with correlation values of 55.56% and 71.54%, respectively. The MMSE and CDR scale scores are the most commonly used psychiatric score scales with high diagnostic accuracy in clinical diagnosis of AD. The ODSS-MRI model scores achieved a high correlation with both, which showed that the neurological function score obtained by ODSS-MRI can reflect the patient’s neurological function level to a certain extent.

### 4.2. Differential Analysis

To evaluate whether the ODSS-MRI exhibited significant differences and the degree of difference between the NC, MCI, and AD groups, Student’s *t*-tests were performed for NC/MCI, MCI/AD, and NC/AD scores, and the *p*-values were shown in Table 3. The results showed that the APOE4 allele, NPI-Q, and GDS scores were not significantly different between NC and MCI groups, which means that they could not distinguish NC from MCI well. The *p*-values of the remaining clinical test indicators were less than 0.0001. The *p*-values of ODSS-MRI between NC/MCI, MCI/AD, and NC/AD groups were much smaller than those of other psychiatric scale scores or indicators, which indicates that the neurological function scores obtained by the ODSS-MRI model exhibited more variability between the groups compared with other indicators and was more effective in discriminating different stages of AD progression. In particular, the ODSS-MRI showed much more variability between MCI/AD and NC/AD groups than other scores, indicating that the ODSS-MRI model scores were significantly different between AD and the other groups.

### 4.3. Sensitivity, Specificity, and Accuracy

In this experiment, the FAQ, MMSE, CDR, and ODSS-MRI were used as classification indicators to calculate the sensitivity, specificity, and accuracy in the two-classification and three-classification tasks, respectively, and the results are shown in Table 4 and Table 5. In the two-classification task of NC/MCI, all four scale scores had high specificity, but the sensitivity of both the FAQ and MMSE was only 20–40%. This indicated that both of them found it difficult to accurately identify MCI patients mixed in NC. In the MCI/AD two-classification task, the specificity of both FAQ and MMSE was low, and the sensitivity of CDR was considerably low (only 1%). This indicated that the CDR scores of AD were mixed with MCI. Although ODSS-MRI did not yield the best results in the sensitivity and specificity experiments, both values were high, and this method yield the highest accuracy (89.42%), approximately 16% higher than the next highest index. In the NC/AD task, all scales performed well because of the simplicity of the classification task. Overall, ODSS-MRI, while performing the best in several tasks, was close to the best-performing indicators in others. Except for the NC/MCI task with 1.79% lower than the CDR scale, the other two tasks achieved the highest accuracy, especially in the accuracy experiment.

In the three-classification task of NC/MCI/AD, the sensitivity of the FAQ and MMSE for MCI was not high and the accuracy was low compared with the CDR and ODSS-MRI, indicating that the FAQ and MMSE are not appropriate for identifying the stages of AD, especially for MCI. The sensitivity of CDR for AD and the specificity of MCI were lower than the other approaches, which indicated that the CDR scale score was significantly weak for the recognition of AD. This is consistent with the characteristics of CDR in two-classification tasks. Compared with other psychiatric rating scales, ODSS-MRI exhibited good sensitivity, specificity, and accuracy in the recognition of NC, MCI, and AD, and there were no obvious defects in the recognition of each stage of AD. The accuracy was slightly lower than CDR in the recognition of NC, and the recognition effect of the other two was the best, especially for MCI recognition accuracy (89.57%), which was much higher than that of the other psychiatric rating scales.

### 4.4. Scatter and Box-Line Plots

**Figure 6:**
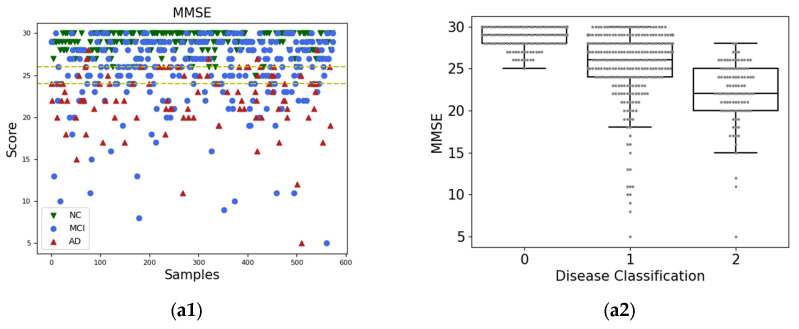

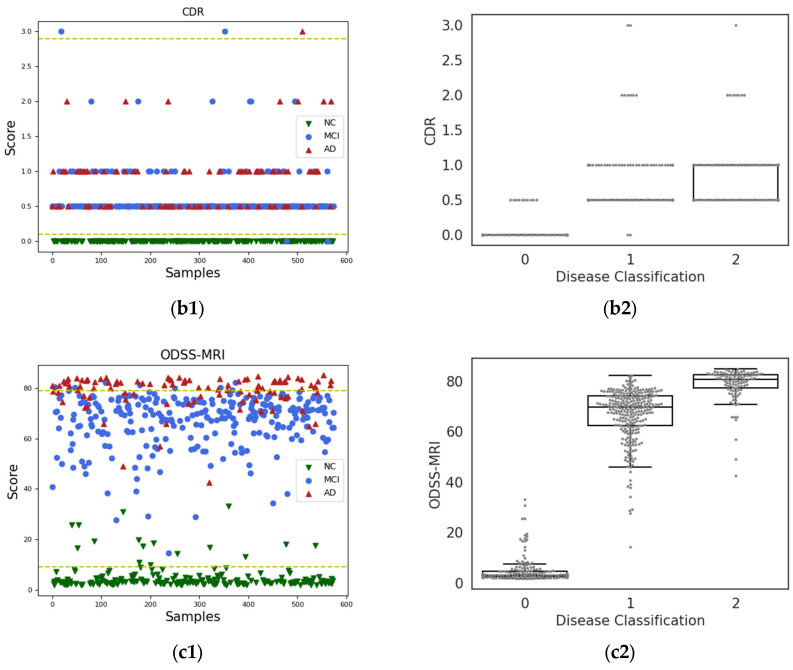
Scatter plots for MMSE (**a1**), CDR (**b1**), and ODSS-MRI (**c1**) and Box-line plots for MMSE (**a2**), CDR (**b2**), ODSS-MRI (**c2**).

In this experiment, to show the distribution of neurological function scores in Alzheimer’s disease patients at each stage, the MMSE, CDR, and ODSS-MRI scores of patients were plotted as scatter plots and box-line plots, as shown in Figure 6. The experimental results showed that the MCI score was distributed in all intervals from the scatter plot. Although a significant difference was observed between the mean value of the MCI group and the mean of the NC and AD groups from the box plot, the main body of the distribution overlapped more with the distribution of the NC and AD groups. This indicated that the MMSE scores were almost indistinguishable for MCI patients. Unlike the MMSE scale scores, the CDR scale scores were able to distinguish NC well, but could not distinguish MCI from AD, while the CDR scores were not sufficiently fine-grained and only divided into seven levels. The ODSS-MRI scores exhibited good differentiation between NC, MCI, and AD compared with MMSE and CDR scores, and the degree of response to subtle changes in neurological level was much higher than that of CDR. Based on the box plot, the mean difference between the three groups was evident, and the data distribution of the subjects overlapped less and was evenly distributed. In summary, for the MMSE and CDR scores, no clear boundaries were observed between groups in each stage of AD, and the CDR scores had fewer levels, which cannot effectively reflect the changes in AD progression. The ODSS-MRI effectively described the subtle changes in neurological function during the AD progression and exhibited better intra-class distribution dispersion. This indicated that the ODSS-MRI was better suitable to describe the AD progression.

### 4.5. Statistical Information Table

To further evaluate the interpretation of AD progression by the ODSS-MRI, we counted statistical information such as variance, interquartile difference, and range of the ODSS-MRI model score after normalization and compared them with the normalized MMSE and CDR scores, and the statistical results are shown in Table 6. The results show that the variance, standard deviation, and quartile deviation of ODSS-MRI are larger than those of MMSE and CDR, and the variation ratio is also higher, which indicates that the dispersion degree of ODSS-MRI scores is higher than that of the other two. The scores are not stacked on a score value, which indicated that ODSS-MRI is better suitable to respond to subtle changes in AD progression.

### 4.6. Pareto Analysis

**Figure 7:**
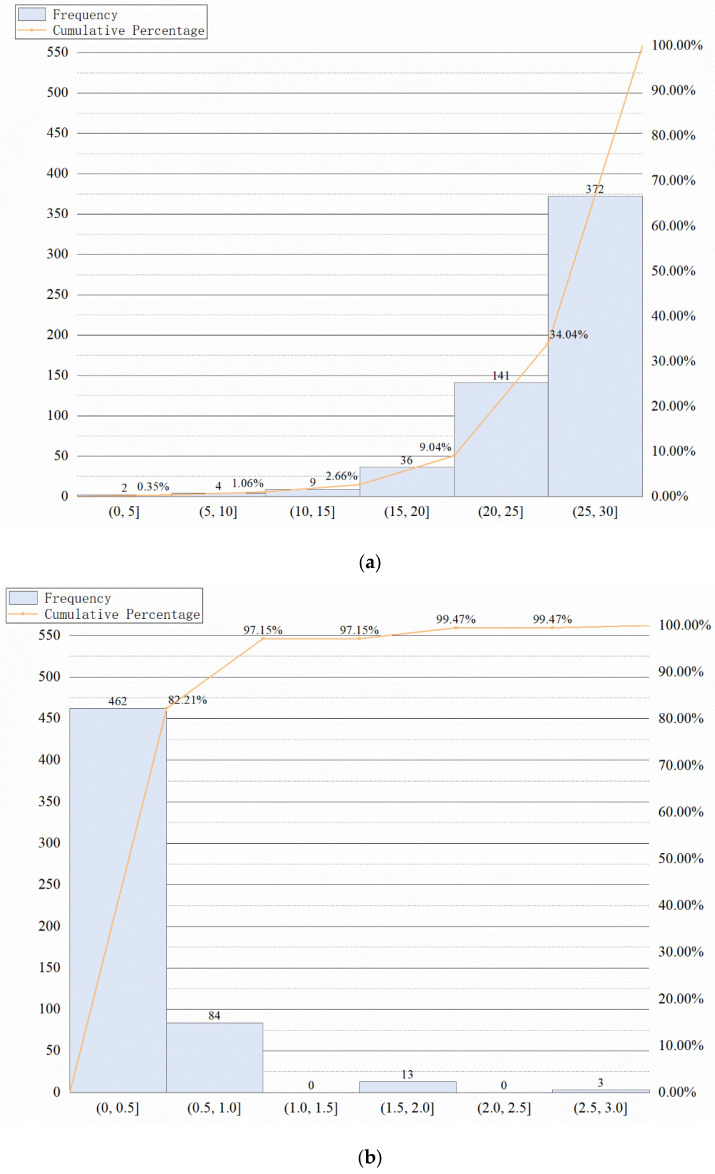

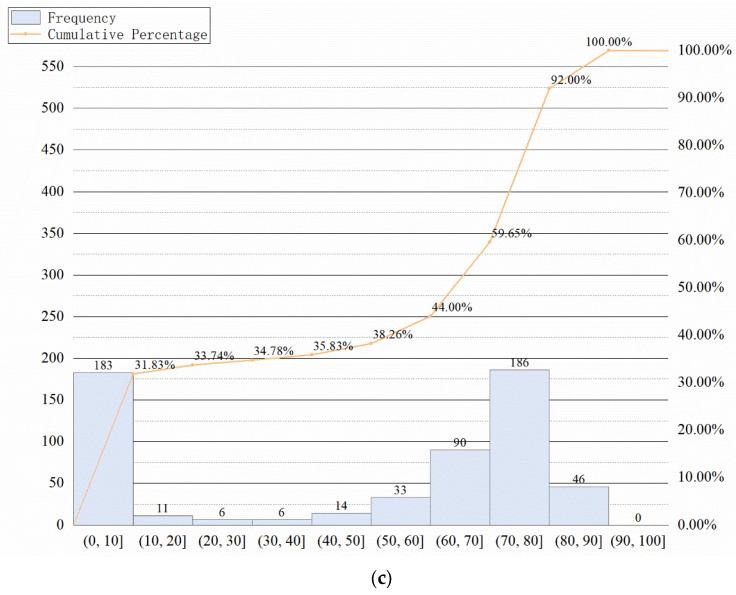
Pareto analysis chart of MMSE (**a**), CDR (**b**), and ODSS-MRI (**c**).

To further illustrate the distribution of the ODSS-MRI, MMSE, and CDR scores, we plotted the Pareto analysis of the three groups, and the results are shown in Figure 7. The figure shows that ODSS-MRI is distributed within each interval and the cumulative curve is relatively smooth. Conversely, in MMSE and CDR, 90% of the score data are concentrated in two intervals and the cumulative curve is considerably steep. This indicates that the MMSE and CDR scores are not sufficiently granular, with severe data accumulation and insufficient refinement for the evaluation of the neurological function of the patient. The experiment showed that the ODSS-MRI scores were more granular compared to the traditional psychiatric rating scales.

## 5. Discussion and Conclusions

In this paper, we proposed a neurological function evaluation model, based on contrastive learning for the evaluation of the progression of AD. Compared with traditional psychiatric rating scales, the data-driven ODSS-MRI ensures objectivity and consistency because it is completely based on medical imaging data and is not affected by biased subjectivity from physicians with different experiences and patients in different statuses during disease severity testing. The neurological function score obtained by the model has a Pearson correlation coefficient of 88.55% and a Spearman correlation coefficient of 87.12% with the stages of AD, which are much higher than those of traditional psychiatric rating scales. This indicates that the ODSS-MRI is superior at describing AD progression. Moreover, the ODSS-MRI exhibits high accuracy in discriminating different progressive stages of AD compared to existing methods, which demonstrated its utility in terms of the neurological function evaluation of AD patients. Above all, this study developed a new method for the neurological function evaluation in AD, which proposed an objective dementia severity scale only based on the whole brain imaging data of patients with contrastive learning and improved the reliability and consistency of existing psychiatric rating scales. This method can potentially be applied to other disease severity evaluation tasks.

## Figures and Tables

**Table 1:** Subject information statistics.

Stage of Disease	AD	MCI	NC
Number	149	263	221
Sex (male/female)	79/70	156/107	115/106
Age	76.3	75.8	77.4
Number of Images	542	1425	1030

**Table 2:** Correlation analysis between different indicators and disease stages.

	Pearson Correlation (%)	Spearman Correlation (%)
APOE4 A1	27.74	28.078
APOE4 A2	33.78	33.89
NPI-Q	40.04	50.59
GDS	23.25	31.96
FAQ	65.73	74.60
MMSE	57.956	68.51
CDR	70.81	85.77
ODSS-MRI	88.55 ± 0.86	87.12 ± 1.3

**Table 3:** The *p*-value of differential analysis using different indicators.

	NC vs. MCI	MCI vs. AD	NC vs. AD
APOE4 A1	$2.06\times 10^{-2}$	$4.13\times 10^{-8}$	$7.43\times 10^{-11}$
APOE4 A2	$3.83\times 10^{-4}$	$7.73\times 10^{-10}$	$2.65\times 10^{-14}$
NPI-Q	$3.57\times 10^{-3}$	$2.31\times 10^{-15}$	$1.94\times 10^{-11}$
GDS	$7.38\times 10^{-1}$	$7.92\times 10^{-8}$	$2.67\times 10^{-5}$
FAQ	$2.14\times 10^{-11}$	$1.69\times 10^{-35}$	$2.22\times 10^{-24}$
MMSE	$1.09\times 10^{-11}$	$2.07\times 10^{-31}$	$1.07\times 10^{-33}$
CDR	$2.73\times 10^{-7}$	$6.08\times 10^{-84}$	$3.53\times 10^{-34}$
ODSS-MRI	$2.52\times 10^{-18}$	$3.40\times 10^{-272}$	$9.03\times 10^{-141}$

**Table 4:** Sensitivity, specificity, and accuracy of evaluation indicators in the two-classification task.

Tasks	Scores	Sensitivity(%)	Specificity(%)	Accuracy(%)
NC/MCI	FAQ	32.09	99.35	68.06
	MMSE	29.63	95.43	63.21
	CDR	99.25	94.87	97.41
	ODSS-MRI	100.00	89.85	95.62
MCI/AD	FAQ	88.33	33.33	50.79
	MMSE	76.09	40.88	55.02
	CDR	1.03	99.25	73.15
	ODSS-MRI	75.73	94.55	89.42
NC/AD	FAQ	91.38	100.00	97.63
	MMSE	93.33	100.00	98.10
	CDR	100.00	100.00	100.00
	ODSS-MRI	100.00	100.00	100.00

**Table 5:** Sensitivity, specificity, and accuracy of evaluation indicators in the three-classification task.

Tasks	Scores	Sensitivity (%)	Specificity (%)	Accuracy (%)
FAQ	AD	81.54	77.01	77.68
	MCI	19.55	96.35	57.86
	NC	99.35	66.32	77.90
MMSE	AD	72.16	82.66	80.85
	MCI	20.74	89.46	56.56
	NC	95.43	62.40	73.94
CDR	AD	1.03	99.57	82.56
	MCI	98.52	63.70	80.43
	NC	94.87	99.46	97.86
ODSS-MRI	AD	75.73	96.82	93.04
	MCI	94.55	85.00	89.57
	NC	89.85	100.00	96.52

**Table 6:** Comparison of dispersion of ODSS-MRI, MMSE, and CDR.

	ODSS-MRI	MMSE	CDR
Variance	0.151101	0.027904	0.022673
Standard deviation	0.388717	0.167044	0.150577
Quartile deviation	0.849023	0.2	0.166667
Variation ratio	0.998261	0.801418	0.510676

## Data Availability

Data used in the preparation of this article were obtained from the Alzheimer’s Disease Neuroimaging Initiative (ADNI) database (adni.loni.usc.edu, accessed on 24 June 2022). As such, the investigators within the ADNI contributed to the design and implementation of ADNI and provided data but did not participate in the analysis or writing of this report. A complete listing of ADNI investigators can be found at http://adni.loni.usc.edu/wp-content/uploads/how_to_apply/ADNI_Acknowledgement_List.pdf, accessed on 24 June 2022.
